# Nonalcoholic Fatty Liver Disease: Correlation of the Liver Parenchyma Fatty Acid with Intravoxel Incoherent Motion MR Imaging–An Experimental Study in a Rat Model

**DOI:** 10.1371/journal.pone.0139874

**Published:** 2015-10-13

**Authors:** Seung-Man Yu, Sung Hwan Ki, Hyeon-Man Baek

**Affiliations:** 1 Department of Biomedical Engineering, Research Institute of Biomedical Engineering, College of Medicine, The Catholic University of Korea, Seoul, Korea; 2 Department of Radiological Science, Gimcheon University, Gimcheon, Gyeongsangbuk-do, Korea; 3 Department of Toxicology, College of pharmacy, Chosun University, Gwangju, Korea; 4 Korea Basic Science Institute, Yeongudanji-Ro, Ochang-eup, Cheongwon-gun, Chungbuk, Korea; Northwestern University Feinberg School of Medicine, UNITED STATES

## Abstract

**Purpose:**

To prospectively evaluate the changes in fatty acid concentration after administrating a 60% high-fat diet to a non-alcoholic fatty liver disease rat model and to perform a correlation analysis between fatty acid with molecular diffusion (D_true_), perfusion-related diffusion (D_fast_), and perfusion fraction (P_fraction_).

**Material and Methods:**

This prospective study was approved by the appropriate ethics committee. Ten male Sprague-Dawley rats were fed a 60% high-fat diet until the study was finished. Point-resolved spectroscopy sequence ^1^H-MRS with TR = 1,500 msec, TE = 35 msec, NEX = 64, and 8×8×8 mm^3^ voxel was used to acquire magnetic resonance spectroscopy (MRS) data. Diffusion-weighted imaging was performed on a two-dimensional multi-b value spin echo planar image with the following parameters: repetition time msec/echo time msec, 4500 /63; field of view, 120×120 msec^2^; matrix, 128×128; section thickness, 3 mm; number of repetition, 8; and multiple b value, 0, 25, 50, 75, 100, 200, 500, 1000 sec/mm^2^. Baseline magnetic resonance imaging and magnetic resonance spectroscopy data (control) were acquired. ^1^H proton MRS and diffusion-weighted imaging were obtained every 2 weeks for 8 weeks. The individual contributions of the true molecular diffusion and the incoherent motions of water molecules in the capillary network to the apparent diffusion changes were estimated using a least-square nonlinear fitting in MatLab. A Wilcoxon signed-rank test with the Kruskal-Wallis test was used to compare each week’s fatty acid mean quantification. Spearman’s correlation coefficient was used to evaluate the correlation between each fatty acid (e.g., total lipid (TL), total saturated fatty acid (TSFA), total unsaturated fatty acid (TUSFA), total unsaturated bond (TUSB), and polyunsaturated bond (PUSB)) and intravoxel incoherent motion (IVIM) mapping images (e.g., D_true_, D_fast_, and P_fraction_).

**Results:**

The highest mean TL value was at week 8 (0.278 ± 0.10) after the administration of the 60% high-fat diet, followed by weeks 6, 4, 2, and 0. The concentration level (16.99±2.29) of TSFA at week 4 was the highest. No significant differences in the concentrations of TUSFA, TUSB, or PUSB were observed in different weeks.

**Conclusion:**

After the administration of the 60% high-fat diet in nonalcoholic fatty liver disease model, TL and TSFA depositions had significant changes. The mean concentrations of TUSFA, TUSB, PUSB did not significantly change. Total unsaturated fatty acid and polyunsaturated bond showed positive correlations with D_true_ and P_fraction_.

## Introduction

Non-alcoholic fatty liver is a common disease that is currently experienced bu 10–24% of the world’s population [1]. It can progress into chronic liver diseases such as liver fibrosis and cirrhosis [2–4]. In the USA, 32–37% of nonalcoholic fatty liver disease (NAFLD) patients progress to liver fibrosis in 3–6 years, with 12% experiencing liver cirrhosis in 8–10 years [5–8]. A simple scoring system for predicting cirrhosis in nonalcoholic fatty liver disease has been used that measured platelet, albumin, and AST/ALT ratios in blood samples [9]. NAFLD is usually associated with metabolic syndromes (MetS), such as type 2 diabetes, insulin resistance, hypertension, and dyslipidemia, that are accompanied by abdominal obesity [10–11]. Accordingly, clinical approaches to investigating the relationship between NAFLD and the prevalence of atherosclerosis and cardiovascular disease have been conducted [12]. NAFLD is not a simple risk factor of type 2 diabetes, cardiovascular disease, or liver cirrhosis.

Liver biopsy has been accepted as the gold standard for diagnosing NAFLD. However, liver biopsy is not only invasive but also clinician-dependent, which often causes sampling errors [13]. Moreover, it is impossible to use this method over a long period to monitor the progress of NAFLD. In a 2014 study, Davide et al. examined whether extracellular vesicles are increased in the liver and blood during experimental NAFLD during searchers for bio-markers [14]. Magnetic resonance spectroscopy (MRS) has been widely used to diagnose NAFLD [15–18]. MRS is a noninvasive and *in vivo* method with high sensitivity and specificity. It is frequently used to diagnose various diseases such as NAFLD, liver fibrosis, and cirrhosis [19–22]. Total lipid (TL), total saturated fatty acid (TSFA), total unsaturated fatty acid (TUSFA), total unsaturated bond (TUSB), and polyunsaturated bond (PUSB) can be calculated with liver fatty acid analysis using ^1^H-MRS through the signal integration of lipid methyl protons (-CH_3,_ 0.9 ppm), methylene protons ((-CH_2_-)n, 1.3 ppm), allylic protons (-CH_2_-C = C-CH_2_-, 2.0 ppm), diallylic protons (= C-CH_2_-C =, 2.8 ppm), and methene protons (-CH = CH-, 5.3 ppm) [19]. Many studies have been conducted on fatty acid deposition in liver parenchyma and fatty acid changes in liver fibrosis using animal models [19, 23].

Diffusion-weighted imaging (DWI) techniques have been recently attempted for diagnosing fatty liver, liver fibrosis, and cirrhosis [24–26]. In particular, the intravoxel incoherent motion (IVIM) is a method of obtaining multi b values to encompass both low-b-value and high-b-value diffusion-weighted images to reflect the random microscopic motion that occurs in voxels on MRI of either intracellular or extracellular water molecules and the micro-circulation of blood through non-linear bi-exponential graph fitting [27]. Previous studies have shown that pure molecular diffusion and perfusion fraction values in the liver parenchyma of liver fibrosis or cirrhosis were lower than those in a control group [24–28]. In fatty liver patients, fat deposition in the liver parenchyma was similar to that in liver fibrosis patients because of distortion and sinus compression of the microcirculatory anatomy and reduced pure molecular diffusion [27–28]. However, the correlation of changes in fatty acid in the liver parenchyma with fat deposition and true diffusion with blood microcirculation has not yet been studied.

Therefore, the objectives of this study were to examine the changes in fatty acid concentration after high-fat diet administration and to determine factors that affect NAFLD through correlation analysis between fatty acid and pure molecular diffusion (D_true_), perfusion-related diffusion (D_fast_), or perfusion fraction (P_fraction_).

## Materials and Methods

This study was carried out in strict accordance with the recommendations in the Guide for the Care and Use of Laboratory Animals of the National Institutes of Health. The protocol was approved by the Committee on the Ethics of Animal Experiments of the Korea Basic Science Institute (KBSI-AEC 1305). All surgery was performed under sodium pentobarbital anesthesia, and all efforts were made to minimize suffering.

### Animal Model

Ten male 8-week-old Sprague-Dawley rats that weighed 100–150 g were housed with ad libitum access to water. The animal care facility was controlled for humidity and temperature on a 12 h light-dark schedule. All Sprague-Dawley rats were fed a 60% high-fat diet that contained 60% fat, 20% protein, and 20% carbohydrate (D12492, Research Diets, New Brunswick, NJ) until the experiment was complete [29]. Baseline MRI and MRS data were acquired before the rats were fed the high-fat diet. MRI and MRS data were also acquired every 2 weeks for 8 weeks according to the schedule of the ^1^H-MRS experiments. The experimental rats were anesthetized for all surgical and imaging procedures by general inhalation anesthesia (isofluorane 1.5 to 2.5% vol., plus O_2_). After completion of the imaging study, mice were sacrificed under deep anesthesia and the livers were excised and processed for further histological analysis.

### *In vivo* Liver ^1^H MRS

All MRI and ^1^H-MRS experiments were performed on a 3.0Tesla MRI scanner (Achiva Tx 3.0 T; Philips Medical Systems, Netherlands) with a maximum gradient of 200 mT/m using a 4-channel animal coil (CG-MUC18-H300-AP, Shanghai Chenguang Medical Technologies Co., Ltd., China). During liver MRS and imaging, all NAFLD model rats were anesthetized with isoflurane/air at 1.0 to 1.5% via a nose cone with respiratory monitoring [30]. Using T_2_-weighted fast spin echo, whole liver parenchyma images were acquired in three transverse axial (FOV 60 mm×60 mm, slice thickness = 1.5 mm), coronal (FOV 6 cm×6 cm, slice thickness = 1.5 mm), and sagittal (FOV 6 cm×6 cm, slice thickness = 1.5 mm) planes to localize voxels or volume of interest for MRS.

**Fig 1:**
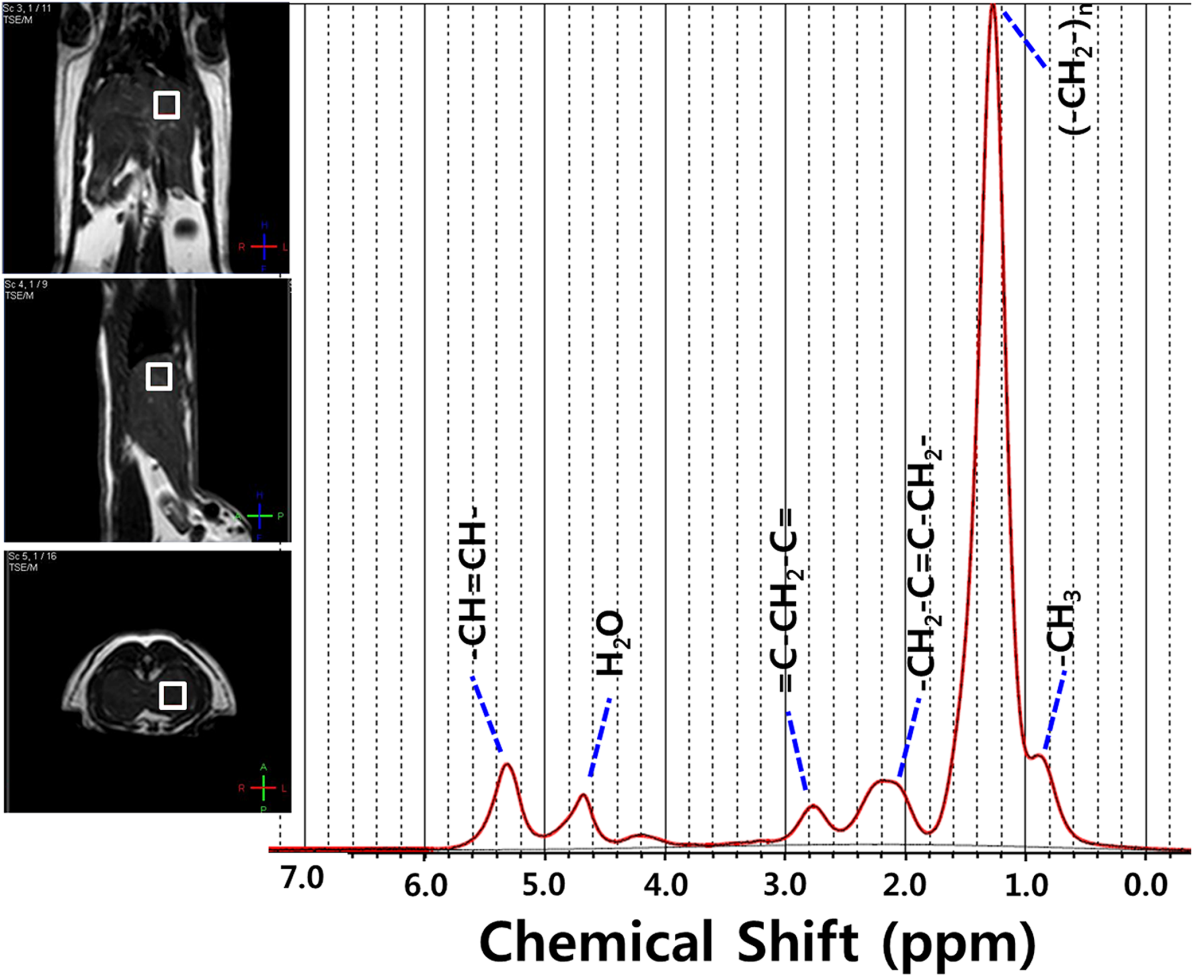
*In-vivo* MRS analysis. Typical liver ^1^H proton magnetic resonance spectroscopy spectra at week 8 after the start of the high-fat diet administration, with typical voxel (0.8×0.8×0.8 cm^3^) placement, were shown in the T_2_-weighed axial, sagittal and coronal turbo spin images. The water (H_2_O) and 4.7 ppm signals were effectively suppressed in the spectra.

We used a point-resolved spectroscopy (PRESS) sequence for localized ^1^H-MRS with TR = 1,500 msec, TE = 35 msec, NEX = 64, and total scan time = 10 minute. An 8×8×8 mm^3^ voxel was placed within a homogeneous liver parenchyma to avoid large blood vessels, as shown in Fig 1.

We applied iterative VOI shim [31]. The water signal of each VOI was suppressed by variable pulse power and optimized relaxation delays before the scan. The signal was shimmed to a line width of lipid (4 to 6 Hz) over VOI (30).

### IVIM MR imaging

**Fig 2:**
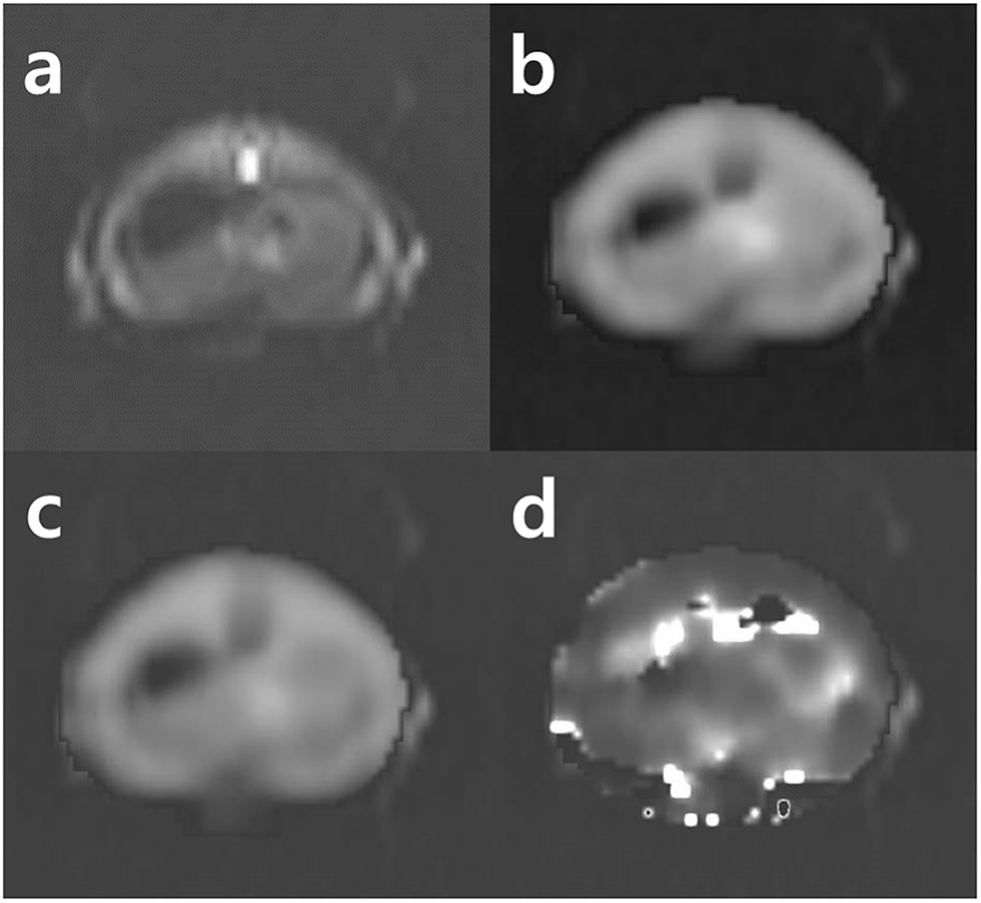
Week 4 NAFLD IVIM data mapping image of rat. (a) Axial diffusion-weighted image of liver (b = 200 sec/mm^2^); (b) D_fast_ mapping image; (c) D_true_ mapping image; (d) P_fraction_ mapping image.

The multi b value DWI was conducted immediately after the ^1^H-MRS data were obtained by using the same MR system. Whole liver parenchyma transverse axial images were obtained. DWI was performed on a two-dimensional multi-b value spin echo planar imaging with the following parameters: prepetition time /echo time, 4500 /63 msec; field of view, 120×120 mm^2^; matrix, 128×128; section thickness, 3 mm; number of repetition, 8; and multiple b value, 0, 25, 50, 75, 100, 200, 500, and 1000 sec/mm^2^. Individual contributions of true molecular diffusion and incoherent motions of water molecules in the capillary network to the apparent diffusion changes, D_true_, D_fast_, and P_fraction_ were estimated as shown in Fig 2 using a least-square nonlinear fitting in MatLab (Mathworks, Natick, MA, USA) by fitting, pixel by pixel, the DWI signal decay in the region of interest (ROI) to the IVIM by-compartmental model as follows: SI/SI_0_ = (1-P_fraction_)×exp (-bD_true_) + P_fraction_×exp (-bD_fast_), where SI was the signal intensity and P_fraction_ was the perfusion fraction linked to blood volume.

### Histology

After 8 weeks of MRI and ^1^H-MRS study, all rats were sacrificed for histological evaluation. One additional normal animal was sacrificed as a control. All liver specimens were fixed in formalin, embedded in paraffin, sectioned, and examined under light microscopy after standard hematoxylin-eosin staining and Prussian blue staining to confirm fat and iron deposition in the liver parenchyma.

### MRS Analysis

Raw ^1^H-MRS data were analyzed using LCModel software (version 6.3-1H, Stephen W. Provencher). Spectrum type with lipid-11 was selected for quantifying all lipids, with water data as references. All lipid peaks were automatically calculated in LCModel. The LC model parameters were set according to the LCModel & LCMgui user’s manual (http://s-provencher.com/pages/lcm-manual.shtml). Less than 10% standard deviation (%SD) of metabolite quantification data was allowed. The %SD called the Cramér-Rao lower bound of useful reliability indicators was used for error estimates. The integrating areas under peaks were measured as follows: signal integrals in lipid methyl protons at 0.90 ppm, methylene proton at 1.30 ppm, allylic protons at 2.02 ppm, diallylic protons at 2.77 ppm, and methane protons at 5.30 ppm. For relative quantification, total lipid ((-CH_2_-)n / noise), total saturated fatty acid, total unsaturated fatty acid, total unsaturated bond, and polyunsaturated bond were quantified by separating each peak area of (-CH_2_-)n, -CH_2_-C = C-CH_2_-, = C-CH_2_-C =, and -CH = CH- by -CH_3_ as shown in Table 1.

**Table 1:** Peak Area Ratios of Various Metabolite Indices Measured by Proton Magnetic Resonance Spectroscopy (^1^H-MRS).

Index	Peak Area Ratio	Frequency
Total lipid	(-CH_2_-)n/water	1.3/4.7 ppm
Total saturated fatty acid	3(-CH_2_-)/2(-CH_3_)	1.3/0.9 ppm
Total unsaturated fatty acid	3(-CH_2_-C = C-CH_2_-)/4(-CH_3_)	2.0/0.9 ppm
Total unsaturated bond	3(-CH = CH-)/2(-CH_3_)	5.3/0.9 ppm
Polyunsaturated bond	3 (= C-CH_2_-C =) /2(-CH_3_)	2.8/0.9 ppm

### IVIM MR Analysis

Extracted mapping imaging diffusion coefficient, pseudo diffusion coefficient, and perfusion fraction were analyzed using standard software on the workstation. All ROIs were manually positioned by one author (Yu). The region of interest of each image was a set circle in the right lobe that was just about the same as the VOI region of MRS. Every ROI size was manipulated differently during measurement to avoid IVIM mapping imaging artifacts. The Wilcoxon signed-rank test with the Kruskal-Wallis test was used to compare each week’s fatty acid mean quantifications with significance level *p*<0.05. Results are expressed as mean ± standard deviation. Spearman’s correlation coefficient was used to evaluate the correlations between each fatty acid (e.g., TL, TSFA, TUSFA, TUSB, and PUSB) and IVIM mapping images (e.g., D_true_, D_fast_, and P_fraction_) with a significance level of *p*<0.05. All statistical analyses were performed using SPSS version 20.0 (SPSS Incorporated, Chicago, IL, USA).

## Results

### Comparison of the mean of hepatic fatty acids

**Fig 3:**
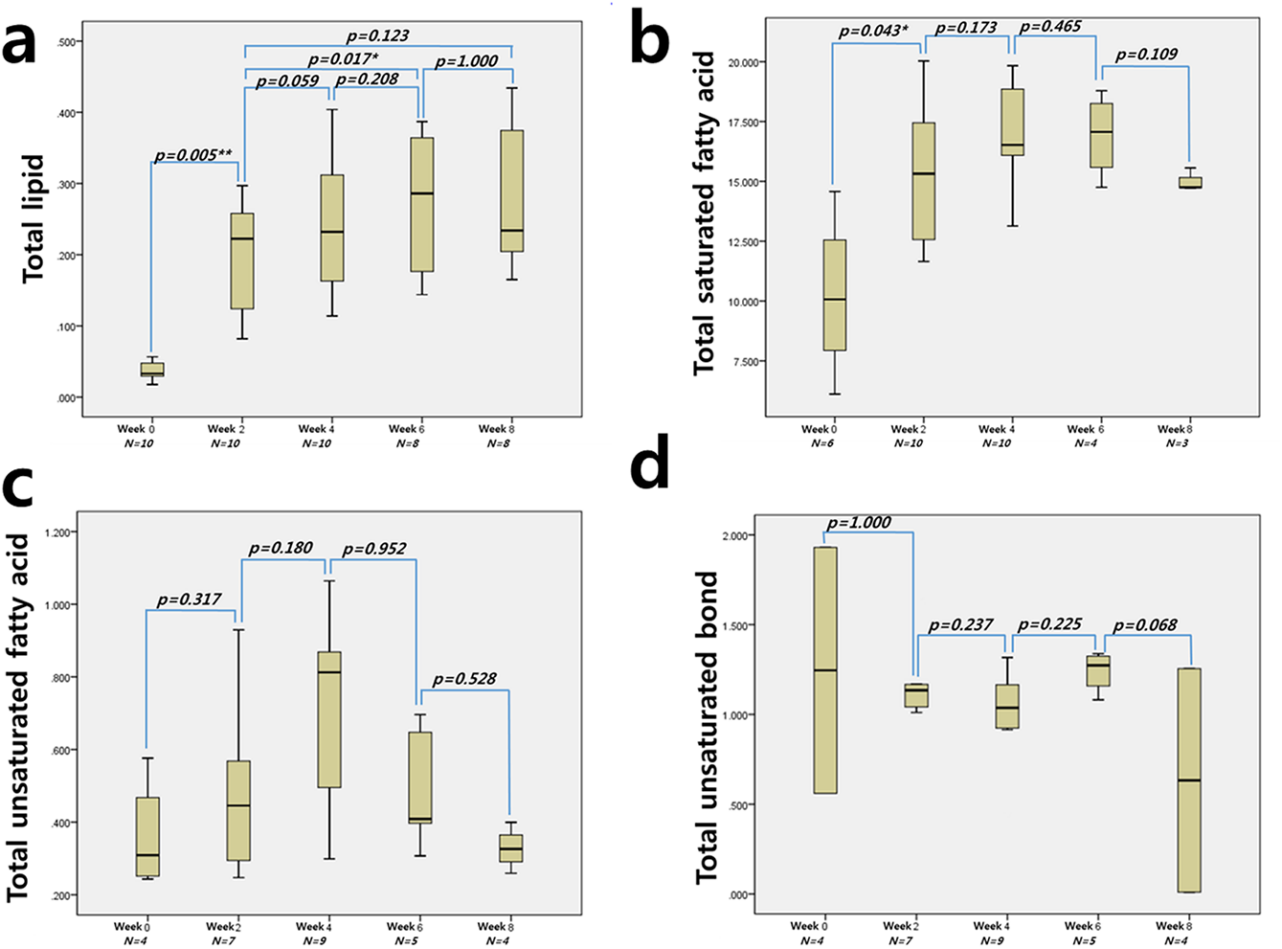
The mean differences from the fatty acid comparisons of each week’s mean quantification. The total lipid and TSFA showed the highest concentrations at weeks 8 and 6, respectively. There were no significant mean differences in TUSFA, TUSB, or PUSB in any of the weeks.

The differences in the N number shown under the X axis (Fig 3) were observed because 10% or lower % SD values were used.

The highest mean TL value was measured at week 8 (0.278±0.10) after the administration of the 60% high-fat diet, followed by weeks 6, 4, 2, and 0. The TL value at week 2 was 0.201±0.07, which was insignificantly (*p*>0.05) lower than that of week 8. The TL value increase at week 2 from baseline week 0 was the most significant (*p*<0.01) among all of the increases. The mean increases in the TL value at weeks 2 and 4 were also significant (*p<*0.05). The TSFA concentration level (16.99±2.29) at week 4 was the highest. A statistically significant mean difference in the levels was observed only between the baseline and week 2 (*p<*0.05). No significant differences in the concentration levels of TUSFA, TUSB, or polyunsaturated bond PUSB were observed among different weeks.

### Correlation between hepatic fatty acid and IVIM mapping images

The results of the non-parametric analysis of the correlations between hepatic fatty acid and IVIM mapping images are summarized in Table 2.

**Table 2:** Spearman correlation analysis between hepatic fatty acid and IVIM data mapping images.

	D_fast_	D_true_	P_fraction_
Total lipid (n = 46)	*0*.*333 (p = 0*.*012* ^*^ *)*	*0*.*238 (p = 0*.*055)*	*0*.*059 (p = 0*.*349)*
otal saturated fatty acid (n = 30)	*0*.*210 (p = 0*.*124)*	*0*.*260 (p = 0*.*082)*	*0*.*194 (p = 0*.*152)*
Total unsaturated fatty acid (n = 28)	*0*.*129 (p = 0*.*252)*	*0*.*535 (p = 0*.*002* ^**^ *)*	*0*.*359 (p = 0*.*030* ^*^ *)*
Total unsaturated bond (n = 22)	*0*.*159 (p = 0*.*240)*	*-0*.*014 (p = 0*.*475)*	*0*.*089 (p = 0*.*347)*
Poly unsaturated bond (n = 31)	*0*.*413 (p = 0*.*010* ^**^ *)*	*0*.*413 (p = 0*.*011* ^*^ *)*	*0*.*346 (p = 0*.*028* ^*^ *)*

**p<0.01

*p<0.05

**Fig 4:**
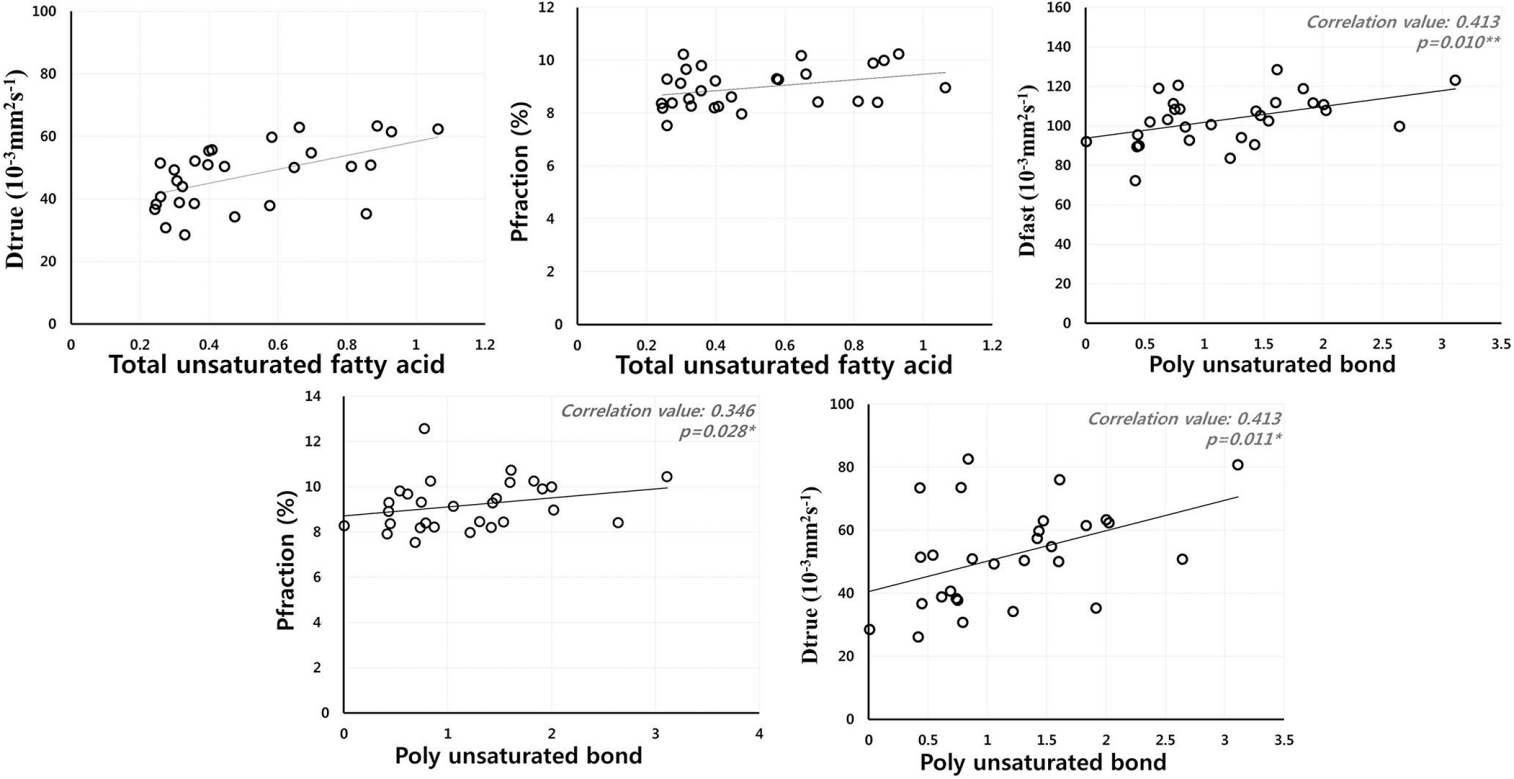
Correlation coefficient values between IVIM mapping data and TUSFA and PUSB. The TUSFA and PUSB had significant correlations with D_true_ and P_fraction_. Blood perfusion TUSB and PUSB in the intravascular compartment with volume fraction (P_fraction_) is described by the pseudorandom blood perfusion (D_fast_), in which the extra vascular compartment is described by the true molecular diffusion (D_true_).

A correlation between total lipid and D_fast_ was observed (*r* = 0.333, p<0.05). P_fraction_ had no correlation with TL. TUSFA showed a positive correlation with D_true._. PUSB was significantly correlated with D_fast_ (*r* = 0.413, p<0.01) and P_fraction_ (*r* = 0.346, p<0.05) in the liver parenchyma, confirming that D_fast_ and P_fraction_ are closely related to liver parenchymal microcirculation. PUSB also correlated with D_true_ (*r* = 0.413, p<0.05, Fig 4).

### Histologic features

**Fig 5:**
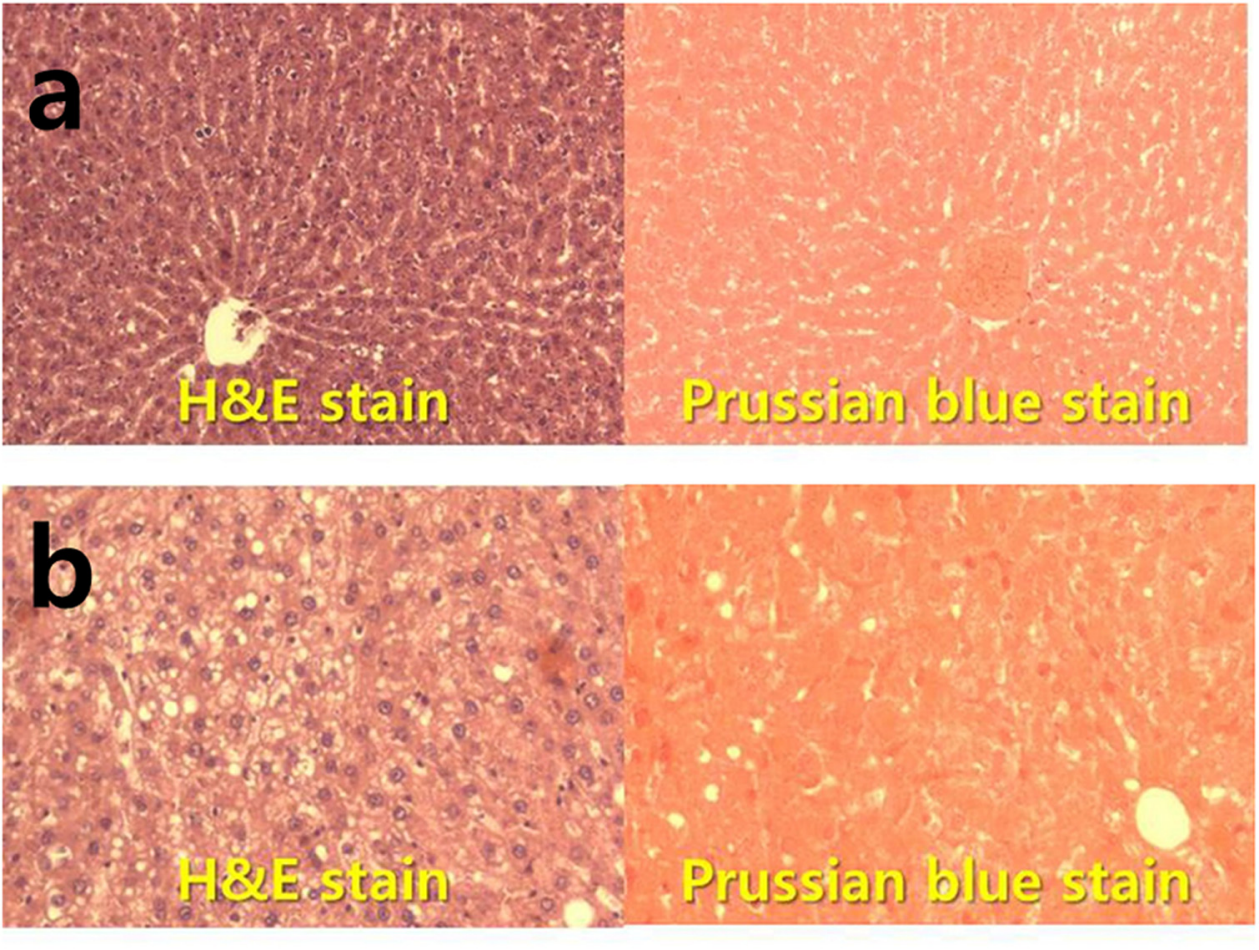
Hematoxylin/eosin and Prussian blue stained histology sections of liver form. (a) Rat not fed with the 60% high-fat diet (×100), (b) nonalcoholic fatty liver rat model fed with the high-fat diet after 8 weeks.

As shown in Fig 5, complete fat deposition in the liver parenchyma was observed in the liver biopsy of a Sprague-Dawley rat through hematoxylin and eosin stain. In the Prussian blue staining observation, iron deposition was not confirmed in any liver tissue.

## Discussion

In this study, changes in fatty acid with fat deposition were observed in liver parenchyma. Correlations among fatty acid, intracellular or extracellular water molecules, and blood microcirculation were investigated. As the NAFLD progresses into chronic liver diseases such as liver fibrosis and cirrhosis, the fat deposition in the liver parenchyma is known to have a negative effect on micro-circulation. In this study, there was great significance in revealing the specific fatty acid through the correlate fatty acid and representing the micro-circulation factor (D_true_, D_fast_, and P_fraction_) in NAFLD.

After the administration of a 60% high-fat diet, the TL level consistently increased, with the peak at week 8. However, the mean difference between week 6 and week 8 was statistically insignificant. TL concentration was saturated at week 6. The NAFLD modeling through the high-fat diet was completed at week 6 and afterwards.

The TSFA concentration calculated through the 1.3 ppm and 0.9 ppm lipid proton ratio increased consistently from week 0 to week 4 and decreased thereafter. The mean difference in TSFA concentrations between week 2 and afterwards was statistically insignificant. The mean levels of TUSFA and PUSB increased from week 0 to week 6 and decreased thereafter. However, the differences were statistically insignificant. The mean differences in TUSB concentrations were also statistically insignificant. Changes in TL and TSFA deposition were the most significant after the administration of the 60% high-fat diet, whereas the changes in the other unsaturated acids and bonds were insignificant. Excellent liver fat deposition was confirmed after hematoxylin-eosin staining for histological evaluation. According to studies by Pacifico et al. [32] and Scorletti et al. [33], the administration of n-3 PUFA positively affected Mets and NAFLD [32–33]. In this study, the long-term administration of the 60% high-fat diet did not affect the changes in PUFA, TUSFA, or TUSB. Therefore, a high-fat diet might be an indirect factor that could adversely affect the liver.

Studies on liver diseases using DWI have been conducted previously [25–28, 34]. Particularly, D_true_, D_fast_, and P_fraction_ data can be analyzed by applying various b-values on the same slice using signal reduction in the low and high b-values through bi-exponential data fitting. In previous studies, a slightly negative correlation between the increase in fat fraction value and the D_true_ value was confirmed with statistical significance [29]. In another study, D_true_ and D_fast_ values were low in the hepatic steatosis group without hepatic steatosis [28]. Based on the results of previous studies, the TL and TSFA were expected to show negative correlations with D_true_ and D_fast_ because the liver fat content calculation from the equation of Guiu et al. (100 ∙ CH_2_)/(CH_2_ + water) was similar to the TL in this study, which was calculated using the 1.3 ppm proton lipid metabolite and water ratios. In this study, no fatty acid showed a correlation with D_true_. Rather, a low correlation was observed between total lipid and D_fast_ (r = 0.333, p<0.05). The etiology in the previous studies that targeted human bodies differed from that in other studies because of fat deposition caused by type *b* and *c* type hepatitis as well as by the fatty liver that was induced by the administration of the high-fat diet. Accordingly, studies on microcirculation in the liver parenchyma according to the type of fatty acid caused by etiology are required. In a 2014 study by Joo et al. that used a NAFLD rabbit model, the D_true_ value in the severe NAFLD group decreased slightly without statistical significance. However, D_fast_ decreased significantly [35]. Because the NAFLD model P_fraction_ reflects the capillary perfusion contribution in ADC, the D_true_ reduction and the D_fast_ increase might have been influenced by the fatting equation that meant a P_fraction_ decrease. The positive correlation of D_true_ with D_fast_ in this study could have been for the same reason. The high D_true_ meant an increase in the pure water diffusivity and in the capillary perfusion [28, 35]. In this study, the fatty acid that showed a positive correlation with D_true_ and P_fraction_ was TUSFA. The correlation coefficient with D_true_ was 0.535 (*p*<0.01), a significantly high correlation. A positive correlation with P_fraction_ was also confirmed with statistical significance. The increase in the pure water diffusivity positively affected capillary perfusion. In particular, PUSB was confirmed to have positive correlations with D_fast_, D_true_, and P_fraction_ with statistical significance. The positive correlation between D_fast_ and P_fraction_ was attributed to the fact that D_fast_ represented the perfusion-related incoherent micro-circulation whereas P_fraction_ represented the blood volume. In a study by Monteiro J et al. [36], n-6 and n-3 polyunsaturated fatty acids had beneficial effects on cardiovascular diseases and non-alcoholic fatty liver diseases. In this study, positive effects on D_fast_, D_true_, and P_fraction_ in USFA and PUSB were confirmed. However, the mean concentrations of USFA and PUSB did not significantly increase after the administration of the 60% high-fat diet. Additional studies on the positive effects of increased TUSFA and PUSB concentrations on blood perfusion in liver parenchyma are merited.

The limitations of this study are as follows: For accurate metabolite quantifications, spin-lattice (T_1_) and spin-spin (T_2_) relaxation must be corrected. T_2_* correction and accurate fat fraction calculation using chemical shift imaging techniques have been attempted not only in MRS but also in multi-echo Dixon [37–41]. In a study by Andrew et al., new methods of *in vivo* T_2_ time measurement through echo-time averaging were suggested [42]. Iron deposition in the liver parenchyma was confirmed to be one of the major factors that triggered T_2_* that was closely correlated with the development of liver fibrosis and cirrhosis [43–45]. In the fatty liver model in this study, iron deposition through Prussian blue staining did not occur at week 8 or afterwards. The absence of T_2_* correction for accurate metabolite quantification was the most significant limitation of this study.

## Conclusions

In conclusion, the administration of the 60% high-fat diet to rats gradually increased the TL and TSFA levels in the liver parenchyma, after which the liver parenchyma became saturated. In the meantime, the levels of TUSFA, TUSFB, and PUSB did not show statistically significant changes. The fatty acids that showed positive correlations with D_true_ and P_fraction_ in the liver parenchyma were TUSFA and PUSB.

## Supporting Information

S1 Dataset The raw data of lipid proton concentration MRS and IVIM parameters. The lipid signals were analyzed using the LCModel algorithm, and a fitting error (the standard deviation) was estimated using the Cramer-Rao lower bounds (CRLB) of the signal amplitude (e.g., at 0.9, 1.3, 2.02, 2.77, and 5.30 ppm). In this work, a CRLB threshold of ≤10% was used for quality control.
(XLSX)

## References

[1] Bataller R, Rombouts K, Altamirano J, Marra F. Fibrosis in alcoholic and nonalcoholic steatohepatitis. Best Pract Res Clin Gastroenterol 2011;25: 231–244. 10.1016/j.bpg.2011.02.010 21497741

[2] Anthony SW, Jude O. Nonalcoholic fatty liver disease and lipids. Curr Opin Lipidol 2012; 23: 345–352. 10.1097/MOL.0b013e3283541cfc 22617751

[3] Stephen AH, Sigurd T, Paul HH. The Natural History of Nonalcoholic Fatty Liver Disease: A Clinical Histopathological Study. Am J Gastroenterol 2003;98: 2042–2047. 14499785 10.1111/j.1572-0241.2003.07659.x

[4] Ivana M, Vesna L, Sanjin R, Sandra M, Branka SM, Lidija O. Nonalcoholic fatty liver disease (NAFLD)–a new factor that interplays between inflammation, malnutrition, and atherosclerosis in elderly hemodialysis patients. Clinical Interventions in Aging 2014;9: 1295–1303 10.2147/CIA.S65382 25143715 PMC4132229

[5] Brent ANT, Stephen HC. Nonalcoholic Steatohepatitis: Summary of an AASLD Single Topic Conference. Hepatology 2003;37: 1202–1219. 12717402 10.1053/jhep.2003.50193

[6] Fassio E, Alvarez E, Domínguez N, Landeira G, Longo C. Natural history of nonalcoholic steatohepatitis: a longitudinal study of repeat liver biopsies. Hepatology 2004;40: 820–826. 15382171 10.1002/hep.20410

[7] Adams LA, Sanderson S, Lindor KD, Angulol P. The histological course of nonalcoholic fatty liver disease: a longitudinal study of 103 patients with sequential liver biopsies. J Hepatol 2005; 42: 132–138. 15629518 10.1016/j.jhep.2004.09.012

[8] Day CP. Natural history of NAFLD: remarkably benign in the absence of cirrhosis. Gastroenterology 2005; 129: 375–378. 16012969 10.1053/j.gastro.2005.05.041

[9] Takaomi K, Yuji O, Masato Y, Kento I, Yoshio E, Hideki F, et al Simple scoring system for predicting cirrhosis in nonalcoholic fatty liver disease. World J Gastroenterol 2014;20: 10108–10114. 10.3748/wjg.v20.i29.10108 25110437 PMC4123339

[10] Ekstedt M, Franzén LE, Mathiesen UL, Thorelius L, Holmqvist M, Bodemar G, et al Long-term follow-up of patients with NAFLD and elevated liver enzymes. Hepatology 2006;44: 865–873. 17006923 10.1002/hep.21327

[11] Rafiq N, Bai C, Fang Y, Srishord M, McCullough A, Gramlich T, et al Long-term follow-up of patients with nonalcoholic fatty liver. Clin Gastroenterol Hepatol 2009;7: 234–238. 10.1016/j.cgh.2008.11.005 19049831

[12] Giovanni T, Guido A. Non-alcoholic fatty liver disease and increased risk of cardiovascular disease. Atherosclerosis 2007;191: 235–240. 16970951 10.1016/j.atherosclerosis.2006.08.021

[13] Brunt EM, Kleiner DE, Wilson LA, Unalp A, Behling CE, lavine JE, et al Portal chronic inflammation in nonalcoholic fatty liver disease (NAFLD): a histologic marker of advanced NAFLD—Clinicopathologic correlations from the Nonalcoholic Steatohepatitis. Clinical Research Network. Hepatology 2009;49: 809–820. 10.1002/hep.22724 19142989 PMC2928479

[14] Povero D, Eguchi A, Li H, Johnson CD, Papouchado BG, Wree A, et al circulating extracellular vesicles with specific proteome and liver microRNAs are potential biomarkers for liver injury in experimental fatty liver disease. PLoS One 9(12): e113651 10.1371/journal.pone.0113651 25470250 PMC4254757

[15] Ren J, Dimitrov I, Sherry D, Malloy CR. Composition of adipose tissue and marrow fat in humans by ^1^H NMR at 7 Tesla. J Lipid Res 2008;49: 2055–2062. 10.1194/jlr.D800010-JLR200 18509197 PMC2515528

[16] Cassidy FH, Yokoo T, Aganivic L, Hanna RF, Bydder M, Middleton MS, et al Fatty liver disease: MR Imaging techniques for the detection and quantification of liver steatosis. Radiograohics 2009;29: 231–260. 10.1148/rg.291075123 19168847

[17] Hwang JH, Bluml S, Leaf A, Ross BD. In vivo characterization of fatty acids in human adipose tissue using natural abundance ^1^H decoupled ^13^C MRS at 1.5 T: clinical applications to dietary therapy. NMR Biomed 2003;16: 160–167. 12884360 10.1002/nbm.824

[18] Strobel K, Hoff J, Pietzsch J. Localized proton magnetic resonance spectroscopy of lipids in adipose tissue at high spatial resolution in mice in vivo. J Lipid Res 2008;49: 473–480. 18024705 10.1194/jlr.D700024-JLR200

[19] Cheung JS, Fan SJ, Gao DS, BEng, Chow AM, Yang J, et al In vivo lipid profiling using proton magnetic resonance spectroscopy in an experimental liver fibrosis model. Acad Radiol 2011;18: 377–383. 10.1016/j.acra.2010.10.012 21167757

[20] Zancanaro C, Nano R, Marchioro C, Sbarbati A, Boicelli A, Osculati F. Magnetic resonance spectroscopy investigations of brown adipose tissue and isolated brown adipocytes. J Lipid Res 1994;35: 2191–2199. 7897317

[21] Venkatesh BA, Lima JAC, Bluemke DA, Lai SL, Steenbergen C, Liu CY. MR proton spectroscopy for myocardial lipid deposition quantification: A quantitative comprarison between 1.5T and 3T. J Magn Reson. Imaging 2012;36: 1222–1230. 10.1002/jmri.23761 22826193 PMC3482140

[22] Runge JH, Bakker PJ, Gaemers IC, Verheil, Hakvoort TBM, Ottenhoff R, et al Quantitative determination of liver triglyceride levels with 3T ^1^H-MR spectroscopy in mice with moderately elevated liver fat content. Acad Radiol 2014;21: 1446–1454. 10.1016/j.acra.2014.06.009 25300722

[23] Corbin IR, Furth EE, Pickup S, Siegelman ES, Delikatny EJ. In vivo assessment of hepatic triglycerides in murine non-alcoholic fatty liver disease using magnetic resonance spectroscopy. Biochim Biophys Acta 2009;1791: 757–763. 10.1016/j.bbalip.2009.02.014 19269347

[24] Bonekamp S, Torbenson MS, Kamel IR. Diffusion-weighted magnetic resonance imaging for the staging of liver fibrosis. J Clin Gastroenterol 2011;45: 885–892. 10.1097/MCG.0b013e318223bd2c 21716125 PMC4337848

[25] Zhou IY, Gao DS, Chow AM, Fan S, Cheung MM, Ling C, et al Effect of diffusion time on liver DWI: An experimental study of normal and fibrotic livers. Magn Reson Med 2014;72: 1389–1396. 10.1002/mrm.25035 24258877

[26] Hope TA, Ohliger MA, Qayyum A. MR imaging of diffuse liver disease: From technique to diagnosis. Radiol Clin N Am 2014;52: 709–724. 10.1016/j.rcl.2014.02.016 24889168

[27] Gulu B, Petit JM, Capitan V, Aho S, Masson D, Lefevre PH, et al Intravoxel incoherent motion diffusion-weighted imaging in nonalcoholic fatty liver disease: A 3.0-T MR study. Radiology 2012;265: 96–103. 22843768 10.1148/radiol.12112478

[28] Yu SM, Kim SS, Paek MY, Goo EH, Ji YS, Choe BY. Correlation between hepatic fat content using 3-echo 3-D Dixon method and intravoxel incoherent motion (IVIM) perfusion MR imaging. Appl Magn Reson 2013;44: 791–801.

[29] Takahashi Y, Soejima Y, Fukusato T. Animal models of nonalcoholic fatty liver disease/ nonalcoholic steatohepatitis. World J Gastroenterol 2012;18: 2300–2308. 10.3748/wjg.v18.i19.2300 22654421 PMC3353364

[30] Cheung JS, Guo H, Leung JCK, Man K, Lai KN, Wu EX. MRI visualization of rodent liver structure and peritoneal adhesion with dialyzate enhancement. Magn Reson Med 2008;59: 1170–1174. 10.1002/mrm.21506 18429030

[31] Tkáč I, Starčuk Z, Choi IY, Gruetter R. In vivo ^1^H NMR Spectroscopy of rat brain at 1 ms echo time. Magn Reson Med 1999;41: 649–656. 10332839 10.1002/(sici)1522-2594(199904)41:4<649::aid-mrm2>3.0.co;2-g

[32] Pacifico L, Giansanti S, Gallozzi A, Chiesa C. Long chain omega-3 polyunsaturated fatty acids in pediatric metabolic syndrome. Mini-Rev Med Chem 2014;14: 791–804. 25307311

[33] Scorletti E, Bhatia L, McCormick KG, Clough GF, Nash K, Calder PC, et al Design and rationale of the WELCOME trial: A randomized placebo controlled study to test the efficacy of purified long chain omega-3 fatty treatment in non-alcoholic fatty liver disease. Contemp clin trials 2014;37: 301–311. 10.1016/j.cct.2014.02.002 24556343

[34] Anderson SW, Soto JA, Milch HN, Ozonoff A, O’Brien M, Hamilton JA, et al Effect of disease progression on liver apparent diffusion coefficient values in a murine model of NASH at 11.7 tesla MRI 2011;33: 882–888. 10.1002/jmri.22481 21448953

[35] Joo IJ, Lee JM, Yoon JH, Jang JJ, Han HK, Choi BI. Nonalcoholic fatty liver disease: Intravoxel incoherent motion diffusion-weighted MR imaging-An experimental study in a rabbit model. Radiology 2014;270: 131–140. 10.1148/radiol.13122506 24091358

[36] Monterio J, Lesle M, Moghadasian MH, Arendt BM, Allard JP, Ma DW. The role of n—6 and n—3 polyunsaturated fatty acids in the manifestation of the metabolic syndrome in cardiovascular disease and non-alcoholic fatty liver disease. Food Funct 2014;5: 426–435. 10.1039/c3fo60551e 24496399

[37] Kühn JP, Hernado D, Mensel B, Krüger PC, Ittermann T, Mayerle J, et al Quantitative chemical shift-encoded MRI is an accurate method to quantify hepatic steatosis. J Magn Reson Imaging 2014;39: 1494–1501. 10.1002/jmri.24289 24123655 PMC4276715

[38] Yokoo T, Shiehmorteza M, Hamilton G, Wolfson T, Schroeder ME, Middleton MS, et al Estimation of hepatic proton-density fat fraction by using MR imaging at 3.0T. Radiology 2011;258: 749–759. 10.1148/radiol.10100659 21212366 PMC3042639

[39] Lee SJ, Yu SM. Effectiveness evaluation of the fat percentage determination in multi-echo T2-corrected single-voxel spectroscopy by comparing the 3-point Dixon and the 6-point interference Dixon technique. Appl Magn Reson 2014;45: 1333–1342.

[40] Meisamy S, Hines CDG, Hamilton G, Sirlin CB, McKenzie CA, Yu H, et al Quantification of hepatic steatosis with T1-independent, T2*-corrected MR imaging with spectral modeling of fat: Blinded comparison with MR spectroscopy. Radiology 2011;258: 767–775. 10.1148/radiol.10100708 21248233 PMC3042638

[41] Lee SS, Lee YJ, Kim NK, Kim SW, Byun JH, Park SH, et al Hepatic fat quantification using chemical shift MR imaging and MR spectroscopy in the presence of hepatic iron deposition: Validation in phantoms and in patients with chronic liver disease J Magn Reson Imaging 2011;33: 1390–1398. 10.1002/jmri.22583 21591008

[42] Prescot AP, Shi X, Choi CH, Renshaw P. In vivo T_2_ relaxation time measurement with echo-time averaging. NMR Biomed 2014;27: 863–869. 10.1002/nbm.3115 24865447 PMC4572890

[43] Sanyal AJ. AGA technical review on nonalcoholic fatty liver disease. Gastroenterology 2002;123: 1705–1725. 12404245 10.1053/gast.2002.36572

[44] Bülow R, Mensel B, Meffert P, Hernando D, Evert M, Kühn JP. Diffusion-weighted magnetic resonance imaging for staging liver fibrosis is less reliable in the presence of fat and iron. Eur Radiol 2013;23: 1281–1287. 10.1007/s00330-012-2700-2 23138385

[45] Shpyleva S, Pogrbna M, Cozart C, Bryant MS, Muskhelishvili L, Tryndyak VP, et al J Nutri Biochem 2014;25: 1235–1242. 10.1016/j.jnutbio.2014.06.012 25256357

